# Enhancing cardiovascular imaging department efficiency through robust quality systems and effective risk management

**DOI:** 10.1093/ehjimp/qyae012

**Published:** 2024-02-16

**Authors:** Roberta Lo Giudice, Alessia Gimelli, Marianna Venditti

**Affiliations:** Imaging Department, Fondazione Toscana Gabriele Monasterio, Via Moruzzi 1, Pisa, Italy; Imaging Department, Fondazione Toscana Gabriele Monasterio, Via Moruzzi 1, Pisa, Italy; Imaging Department, Fondazione Toscana Gabriele Monasterio, Via Moruzzi 1, Pisa, Italy

## Introduction

The cardiovascular imaging landscape has witnessed remarkable advancements, allowing for more precise diagnoses and targeted treatments. One crucial aspect contributing to the success of cardiovascular imaging is the implementation of a robust quality system and effective risk management. To support this concept, this e-letter delves into the findings of a recent study assessing the appropriateness of referrals to a nuclear cardiology laboratory for stress myocardial perfusion imaging (MPI) and highlights the profound impact of a healthy quality system on patient outcomes and resource utilization.^1^

The study evaluated 1870 consecutive patients undergoing MPI. The assessment considered the appropriateness of imaging test referrals based on the 2023 Appropriate Use Criteria and the European Society of Cardiology (ESC) guidelines for chronic coronary syndromes.^2^ Notably, 88% of MPI tests were classified as ‘appropriate’, reinforcing the importance of adherence to established criteria in clinical decision-making. Moreover, appropriate referrals were associated with a higher incidence of moderate-to-severe ischaemia, highlighting the diagnostic accuracy and efficacy of the MPI in cases where the test was deemed appropriate. In contrast, inappropriate and uncertain referrals demonstrated lower rates of ischaemia, emphasizing the importance of proper test indications in obtaining meaningful results. The adoption of ESC guidelines further strengthened the case for quality in the referral process.^2^ Patients managed by guidelines showed a significantly higher prevalence of obstructive coronary artery disease during invasive coronary angiography. This aligns with the broader concept that appropriateness in test referrals leads to better downstream resource utilization, promoting optimal patient care.^3^

## Risk management and patient outcomes

A well-structured quality system not only ensures appropriateness in test referrals but also plays a pivotal role in risk management. The study indicates that adherent management to the international guidelines, which should ideally be linked to the most advanced clinical and pharmacological trials, resulted in a higher likelihood of coronary revascularization compared with non-adherent cases. This implies that when clinical decisions are guided by established protocols grounded in the latest research, the chances of identifying and addressing significant cardiovascular issues are significantly improved, as demonstrated by the EURECA registry.^4^

## The importance of continuous quality improvement

The findings underscore the need for continuous quality improvement in cardiovascular imaging departments, emphasizing the crucial role of the established health quality system and the imperative to adhere diligently to standard operating procedures. Regular evaluations of appropriateness criteria and guideline adherence can identify areas for enhancement, contributing to the evolution of best practices. Furthermore, ongoing training and education for healthcare professionals involved in the referral process are essential to maintaining a high standard of care. Finally, a key aspect of quality improvement is the emphasis on data collection and analysis in each cardiac imaging department. By leveraging data, cardiac imaging departments can identify trends, measure performance metrics, and implement targeted improvements. This data-driven approach empowers healthcare providers to make informed decisions, fostering a culture of continuous learning and improvement.

## Resource optimization and cost-efficiency

Beyond the immediate impact on patient outcomes, a healthy quality system and effective risk management contribute to resource optimization and cost-efficiency. Appropriate test referrals minimize unnecessary procedures, reducing the strain on healthcare resources and mitigating financial burdens on both patients and healthcare systems. In contrast, inappropriate referrals can lead to unnecessary tests and procedures, adding to the overall healthcare expenditure without commensurate benefits.

## Conclusion

The study on the appropriateness of referrals to a nuclear cardiology laboratory sheds light on the far-reaching implications of a well-established quality system and effective risk management in cardiovascular imaging departments.^1^ The high rate of appropriate MPI tests is not only indicative of the current state of quality in clinical decision-making but also emphasizes the potential for continuous improvement.

As we navigate the evolving landscape of healthcare, it is imperative to recognize the pivotal role that quality systems and risk management play in shaping patient outcomes, resource utilization, and the overall efficiency of cardiovascular imaging departments. Embracing a culture of continuous improvement ensures that these departments remain at the forefront of delivering high-quality, patient-centred care.

**Conflict of interest:** R.L.G., A.G., and M.V. have nothing to disclose.

## Data Availability

No new data were generated or analysed in support of this research.

## Lead author biography

Dr Alessia Gimelli is a cardiologist and nuclear medicine specialist. She is the Director of the Health Quality System at Fondazione Toscana G. Monasterio, Pisa, Italy. Dr Gimelli has deep experience in clinical research on cardiovascular diseases and cardiovascular imaging and has contributed to several international high-profile projects. She is the Editor-in-Chief of *European Heart Journal—Imaging Methods and Practice Journal*.
